# Twenty-four-hour mechanical power variation rate is associated with mortality among critically ill patients with acute respiratory failure: a retrospective cohort study

**DOI:** 10.1186/s12890-021-01691-4

**Published:** 2021-10-25

**Authors:** Yi Chi, Qing Zhang, Siyi Yuan, Zhanqi Zhao, Yun Long, Huaiwu He

**Affiliations:** 1 State Key Laboratory of Complex Severe and Rare Disease, Department of Critical Care Medicine, Peking Union Medical College Hospital, Peking Union Medical College, Chinese Academy of Medical Sciences, Beijing, China; 2 Institute of Technical Medicine, Furtwangen University, Villingen-Schwenningen, Germany; 3 Department of Biomedical Engineering, Fourth Military Medical University, Xi’an, China

**Keywords:** Mechanical power, Mortality, Prognosis, Acute respiratory failure

## Abstract

**Objectives:**

Defined as the energy applied to the respiratory system by ventilator, mechanical power (MP) of ventilation reflects the risk of ventilation-induced lung injury. This study aims to explore the relationship between dynamic changes in MP and prognosis in critically ill patients.

**Methods:**

This was a single-centre retrospective cohort study. Patients receiving mechanical ventilation with acute respiratory failure (ARF) and MP > 10 J/min on admission in the ICU were included. MP (J/min) was calculated as 0.098 × minute ventilation (L/min) × [(peak inspiratory pressure + positive end-expiratory pressure)/2] and the MP variation rate (%) as ([baseline MP − 24-h MP]/baseline MP) × 100. Patients were divided into two groups according to whether MP decreased 24 h after admission (MP-improved group defined as 24-h MP variation rate > 0% vs. MP-worsened group defined as 24-h MP variation rate ≤ 0%).

**Results:**

In total, 14,463 patients were screened between January 2015 and June 2020, and finally, a study cohort of 602 patients was obtained. The MP-improved group had a lower ICU mortality rate than the MP-worsened group (24% vs. 36%; *p* = 0.005). The 24-h MP variation rate was associated with ICU mortality after adjusting for confounders (odds ratio, 0.906 [95% CI 0.833–0.985]; *p* = 0.021), while baseline MP (*p* = 0.909) and 24-h MP (*p* = 0.059) were not. All MP components improved in the MP-improved group, while minute ventilation and positive end-expiratory pressure contributed to the increase in MP in the MP-worsened group.

**Conclusions:**

The 24-h MP variation rate was an independent risk factor for ICU mortality among ARF patients with elevated MP. Early decreases in MP may provide prognostic benefits in this population.

**Supplementary Information:**

The online version contains supplementary material available at 10.1186/s12890-021-01691-4.

## Introduction

Critically ill patients with acute respiratory failure (ARF) are usually exposed to high-intensity mechanical ventilation, making them prone to ventilator-induced lung injury (VILI) [1]. Ventilation parameters, including tidal volume [2], driving pressure [3], and mean airway pressure [4], have been shown to correlate with patient outcomes in the intensive care unit (ICU). Among them, the mechanical power (MP) of ventilation has been proposed as a comprehensive index that combines volume, respiratory rate and airway pressure to reflect the intensity of ventilation [5]. MP is the total energy needed to overcome the resistance of a patient’s respiratory system over a period, usually expressed as joules per minute (J/min). Several retrospective studies have indicated that MP is an independent risk factor for predicting short- or long-term outcomes among critically ill patients [6–9]. To our knowledge, however, studies have usually focused only on the association between baseline MP and prognosis. Very limited evidence exists regarding the dynamic change in MP and patient outcomes in the ICU. As both injurious MP and exposure time are considered essential components of VILI development [10, 11], a quick reduction in injurious power over time should provide beneficial effects in terms of patient outcomes.

The aim of our study was to explore the correlation between the 24-h change in MP and the outcomes of ARF patients with elevated MP on ICU admission. We also intend to investigate the relevant factors behind MP changes, namely, the components of MP, including minute ventilation, positive end-expiratory pressure (PEEP) and respiratory compliance, to find possible effective targets for reducing MP.

## Methods

### Study design and settings

This is a single-centre retrospective cohort study performed in the Department of Critical Care Medicine of Peking Union Medical College Hospital, a tertiary hospital with 2000 beds receiving more than 110,000 in-patients per year. Approximately 2500 patients, both medical and surgical, are admitted to the ICU each year.

### Study population

Patients with ARF, defined as the ratio of partial pressure of oxygen (PaO_2_) to fraction of inspired oxygen (FiO_2_) less than 300 mmHg on admission, who received invasive mechanical ventilation for more than 48 h between 1 January 2015 and 30 June 2020 were eligible for study inclusion. We excluded patients who (1) received treatment with extracorporeal life support; (2) were aged less than 18 years; or (3) had MP ≤ 10 J/min on admission (with a possible lower risk of lung injury [12]). This study was approved by the Institutional Research and Ethics Committee of Peking Union Medical College Hospital.

### Ventilation strategy, MP and MP variation rate

Lung protective strategies were adopted for all patients admitted to the ICU according to the up-to-date guidelines [13–15]. Further, quality control targets were set to guide ventilator settings [16]. Specifically, the initial tidal volume was set at 6–8 ml/kg predicted body weight (PBW) using volume- or pressure-controlled mode. Tidal volume and respiratory rate were adjusted according to arterial blood gas with PaCO_2_ no less than 40 mmHg to avoid unnecessary ventilation. Appropriate sedation and analgesia were applied to achieve this target. If vigorous spontaneous effort was detected despite adequate sedation and analgesia, neuromuscular blocking agents (NMBA) would be prescribed by the responsible attending intensivist. Initial PEEP and FiO_2_ were set based on the lower PEEP-FiO_2_ table [2]. After the patient became relatively stable in hemodynamics, subsequent PEEP was titrated through decremental PEEP trial (started from 15 to 20 cmH_2_O, with 3 cmH_2_O as a step, 3–5 min for each step). The optimal PEEP was set by the maximal static respiratory compliance (tidal volume divided by driving pressure) in some patients and by bedside ultrasound (reaeration score to assess lung recruitment) [17] or electrical impedance tomography (the combination of lowest overdistension and collapse) in others [18]. SpO_2_ was not allowed to reach 100% unless at room air. The ventilator was switched to pressure support mode considering the following criteria: hemodynamic stability, PaO_2_/FiO_2_ ≥ 200 mmHg, PEEP no more than 5 cmH_2_O and some improvement in the underlying condition that caused the respiratory failure [19].

We calculated the dynamic driving pressure as the peak inspiratory pressure minus the PEEP because the plateau pressure was not regularly recorded in our database. Then, MP was calculated as 0.098 × respiratory rate × tidal volume × (peak inspiratory pressure – (0.5 × dynamic driving pressure)), which was equal to 0.098 × minute ventilation (L/min) × [(peak inspiratory pressure + positive end-expiratory pressure)/2][12]. The 24-h MP variation rate (%) was defined as ([baseline MP − 24-h MP]/baseline MP) × 100. The dynamic compliance of the respiratory system (mL/cmH_2_O) was equal to the tidal volume/dynamic driving pressure.

### Data collection

All the clinical data were extracted from the Critical Care Monitor System in Peking Union Medical College Hospital, which recorded real-time clinical data hourly with bedside equipment. To minimize errors, baseline respiratory mechanics and hemodynamic data used in the statistical analyses were an average of the values collected during the first 12 h after ICU admission, and the 24-h corresponding values were an average of the 24 to 36-h values. The primary clinical outcome of this study was ICU mortality. The number of ventilator-free days (defined as the number of days from successful weaning to day 28, with patients who died before weaning being deemed to have no ventilator-free days), length of ICU stay and length of hospital stay were also measured as secondary outcomes. We evaluated the predictive values of baseline MP, 24-h MP and the MP variation rate for ICU mortality rates.

### Statistical analysis

Descriptive data are expressed as numbers and percentages for categorical variables and means (SDs) or medians (IQRs) for continuous variables. Categorical variables were compared using the Pearson chi-square test, whereas continuous variables distributed nonparametrically between groups were compared using the Mann–Whitney U test. Baseline and 24-h values of the same parameter were compared using the Wilcoxon signed-rank test. A competing risk model was used to evaluate the effect of the MP decrease on the probability of successful discontinuation of mechanical ventilation, with death as a time-dependent competing event. Comparisons were performed using the Gray test.

Multivariate logistic regression model was established to determine the effects of baseline MP, 24-h MP and the MP variation rate for ICU mortality by forward stepwise selection procedure; the model was adjusted for age, sex, Acute Physiology and Chronic Health Evaluation II score, admission type, PaO_2_/FiO_2_ ratio and ventilatory ratio on admission (minute ventilation [mL/min] × PaCO_2_ [mmHg])/(PBW [kg] × 100 [mL/min] × 37.5 [mmHg]) [20], prone positioning and neuromuscular blocking agent treatments within 24 h. Variables introduced in this model were either statistically different between MP-worsened and MP-improved groups or considered clinically associated with ICU mortality. The performance of baseline MP, 24-h MP and the MP variation rate were evaluated using the area under the curve of the receiver operating characteristic (AUC-ROC). The differences in AUCs-ROC were analysed by using DeLong’s method.

We performed multiple imputation to account for missing data (shown in Additional file 1: eTable 1) by generating five imputed datasets for the study population [21] and then repeated the model analyses using these imputed datasets to examine the robustness of the findings from the complete case analysis. All comparisons were two-tailed, and a *p* value of less than 0.05 was considered statistically significant. Statistical analyses were performed using SPSS for Windows version 25.0 (SPSS, Chicago, IL).

## Results

### Patient characteristics

**Fig. 1:**
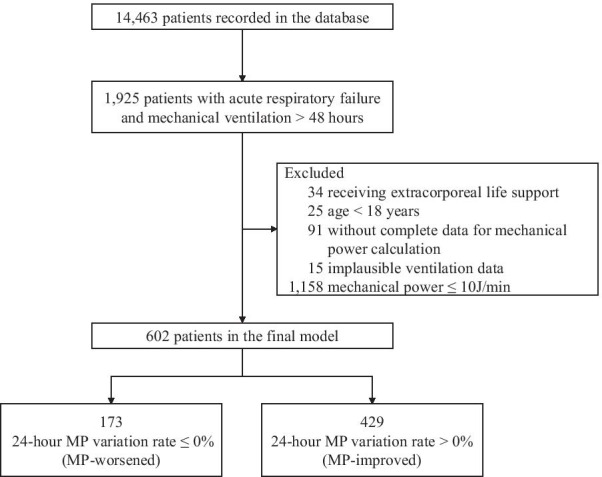
A flowchart of patient inclusion and exclusion

During the study period (January 2015 to June 2020), 14,463 patients were recorded in the database, from which 1,925 patients with ARF received mechanical ventilation for more than 48 h. We excluded 34 patients receiving extracorporeal life support during the first day on admission in the ICU, 25 patients with age less than 18 years, 91 patients without complete data for the calculation of MP and another 15 with implausible ventilation data. At the last step, 1,158 patients with MP no more than 10 J/min were excluded, resulting in a cohort of 602 patients (Fig. 1).

The patients had a median age of 61 years (IQR 50–70), and 155 (26%) of them were women. Overall, 436 patients survived, and 166 patients expired, yielding an ICU mortality rate of 28%. These patients were divided into the MP-worsened group (24-h MP variation rate ≤ 0%) and the MP-improved group (24-h MP variation rate > 0%). The clinical characteristics of the study population are shown in Table 1. Overall, more prone positioning and NMBAs were used in the MP-worsened group.

**Table 1:** Baseline and clinical characteristics of the study population

Variables	Total (n = 602)	MP variation rate ≤ 0% (MP-worsened, n = 173)	MP variation rate > 0% (MP-improved, n = 429)	*p*
Age, years	61 (50, 70)	62 (50, 70)	61 (50, 69)	0.500
Female, n (%)	155 (26)	33 (19)	122 (28)	0.023
Height, cm	170 (165, 175)	172 (165, 175)	170 (163, 175)	0.022
Weight, kg	74 (65, 80)	75 (67, 85)	70 (63, 80)	0.003
Admission type, n (%)				0.425
Medical	251 (42)	77 (45)	174 (41)	
Surgical	351 (58)	96 (55)	255 (59)	
APACHE II score	20 (15, 26)	21 (16, 28)	20 (15, 25)	0.076
SOFA score	10 (5, 13)	10 (5, 14)	10 (6, 13)	0.608
Norepinephrine dose, µg/kg/min	0.26 (0.10, 0.56)	0.27 (0.11, 0.60)	0.24 (0.10, 0.53)	0.368
Respiratory treatment				
Prone positioning, n (%)	178 (30)	68 (39)	110 (26)	0.001
NMBA, n (%)	54 (9)	28 (16)	26 (6)	< 0.001
Ventilation mode				0.231
Volume control, n (%)	549 (91)	154 (89)	395 (92)	
Pressure control, n (%)	53 (9)	19 (11)	34 (8)	
Baseline MP, J/min	12.1 (10.8, 14.1)	11.7 (10.8, 13.8)	12.2 (10.9, 14.3)	0.088
24-h MP, J/min	10.7 (8.9, 13.3)	14.2 (12.4, 16.4)	9.7 (8.4, 11.3)	< 0.001
MP variation rate, %	14 (− 3, 27)	− 14 (− 26, − 6)	21 (12, 31)	< 0.001
ICU length of stay, days	9 (5, 15)	9 (5, 15)	9 (5, 15)	0.731
Hospital length of stay, days	15 (10, 27)	13 (9, 29)	16 (10, 27)	0.065
VFD at day 28, days	16 (0, 22)	10 (0, 21)	16 (0, 22)	0.007
ICU mortality, n (%)	166 (28)	62 (36)	104 (24)	0.005

Data are presented as median (IQR) unless otherwise specified

APACHE, Acute Physiology and Chronic Health Evaluation; SOFA, Sequential Organ Failure Assessment; NMBA, neuromuscular blocking agent; MP, mechanical power; ICU, intensive care unit; VFD, ventilator-free day

### MP and MP variation rate

**Fig. 2:**
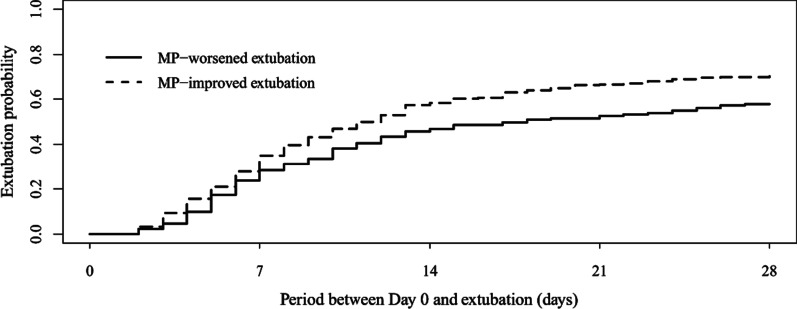
Cumulative incidence curves of discontinuation of mechanical ventilation over time (Gray-test *p* = 0.0026) with death as competing event according to the change of mechanical power (MP)

Baseline MP levels between the MP-worsened group and MP-improved group were similar (11.7 J/min [IQR 10.8–13.8 J/min] vs. 12.2 J/min [IQR 10.9–14.3 J/min]; *p* = 0.088). The 24-h MP variation rates were − 14% [IQR − 26 to − 6%] and 30% [IQR 26–40%], respectively, resulting in a different MP at 24 h (14.2 J/min [IQR 12.4–16.4 J/min] vs. 9.7 J/min [IQR 8.4–11.3 J/min]; *p* < 0.001). Lower ICU mortality rates (36% vs. 24%; *p* = 0.005) and more ventilator-free days at day 28 (10 days [IQR 0–21 days] vs. 16 days [IQR 0–22 days]; *p* < 0.001) were found in the MP-improved group (Table 1). Figure 2 shows the result of the competing risk analysis for the time to discontinuation from mechanical ventilation, with death as the competing event. The MP-improved group was associated with a reduced proportion of patients requiring invasive mechanical ventilation at day 28 (29% vs. 42%; Gray test *p* = 0.0026).

Baseline MP, 24-h MP and the MP variation rate were all associated with ICU mortality in the univariate logistic regression model (Table 2). After adjustments were made for confounders, the 24-h MP variation rate was the only risk factor among them. (odds ratio per 10% increase, 0.906 [95% CI 0.833–0.985]; *p* = 0.021). The results for other variables included in the model are listed in Additional file 1: eTable 2. The AUC-ROC values for baseline MP, 24-h MP and the MP variation rate for predicting ICU mortality were 0.595 (95% CI 0.554–0.634; *p* < 0.001), 0.630 (95% CI 0.590–0.669; *p* < 0.001) and 0.584 (95% CI 0.543–0.623; *p* < 0.001), respectively (Table 3). Based on the maximal Youden index, a cut-off value of ≤ 19% for the MP variation rate would have a sensitivity of 71%, specificity of 43%, positive predictive value of 32% and negative predictive value of 80% for predicting ICU mortality.

**Table 2:** Univariate and multivariate logistic regression analysis for ICU mortality

Variables	Univariate logistic regression		Multivariate logistic regression*	
	Odds ratio (95% CI)	*p*	Odds ratio (95% CI)	*p*
Baseline MP > 10 J/min (n = 602)				
Baseline MP	1.077 (1.027–1.128)	0.002	–	0.909
MP variation rate (per 10% increase)	0.884 (0.818–0.955)	0.002	0.906 (0.833–0.985)	0.021
24-h MP	1.115 (1.063–1.170)	< 0.001	–	0.059
Baseline MP > 15 J/min (n = 107)				
Baseline MP	0.923 (0.800–1.064)	0.270	–	0.234
MP variation rate (per 10% increase)	0.735 (0.605–0.892)	0.002	0.722 (0.584–0.892)	0.003
24-h MP	1.086 (1.001–1.178)	0.048	–	0.210

^*^The odds ratio and 95% confidence intervals (CI) for each variable were calculated after multivariable adjustment for age, sex, Acute Physiology and Chronic Health Evaluation II score, admission type, PaO_2_/FiO_2_ ratio and ventilatory ratio on admission, prone positioning and neuromuscular blocking agent treatments within 24 h

**Table 3:** AUC-ROC of baseline mechanical power, 24-h MP and power variation rate for ICU mortality in different populations

Population	Number of patients	ICU mortality, n (%)	APACHE II score	AUC-ROC (95% CI) for baseline MP	AUC-ROC (95% CI) for 24-h MP	AUC-ROC (95% CI) for 24-h MP variation rate
Unselected ICU patients	8597	405 (4.7)	13 (10, 17)	0.752 (0.743–0.761)	–	–
ARF at baseline with mechanical ventilation > 48 h	1925	322 (16.7)	18 (13, 23)	0.634 (0.612–0.655)	0.613 (0.591–0.635)	0.508 (0.485–0.531)*
ARF with baseline MP > 10 J/min	602	166 (27.8)	20 (15, 26)	0.595 (0.554–0.634)	0.630 (0.590–0.669)	0.584 (0.543–0.623)
ARF with baseline MP > 15 J/min	107	43 (40.2)	23 (18, 30)	0.526 (0.427–0.623)	0.683 (0.586–0.769)	0.683 (0.586–0.770)*

AUC-ROC, areas under curve of receiver operating characteristic; CI, confidence interval; ICU, intensive care unit; ARF, acute respiratory failure; MP, mechanical power

^*^p < 0.05 compared with AUC-ROC of baseline MP as reference by DeLong’s test

Analysis was performed to compare the prognostic values of baseline MP, 24-h MP and the MP variation rate for ICU mortality rate among patients with different baseline MP values in our database (Tables 2 and 3). The AUC-ROC of baseline MP tended to decrease in patients with higher baseline MP, while the AUC-ROC values of 24-h MP and the MP variation rate tended to increase. For 107 patients with baseline MP > 15 J/min, the AUC-ROC of the 24-h MP variation rate was higher than that of baseline MP (*p* = 0.046 by DeLong’s test), and the 24-h MP variation rate remained a risk factor in the multivariate logistic regression model (odds ratio per 10% increase, 0.722 [95% CI 0.584–0.892]; *p* = 0.003).

### MP components

As shown in Table 4, the baseline tidal volume was lower in the MP-worsened group (6.7 mL/kg [IQR 5.9–7.4 mL/kg] vs. 7.1 mL/kg [IQR 6.4–7.9 mL/kg]; *p* < 0.001); however, the 24-h minute ventilation was found to be higher in this group (9.0 L/min [IQR 8.1–10.2 L/min] vs 7.5 L/min [IQR 6.5–8.5 L/min]; *p* < 0.001). PEEP and dynamic compliance of the respiratory system did not differ between groups at baseline (*p* = 0.353; *p* = 0.63). They both improved in the MP-improved group, while PEEP was slightly elevated in the MP-worsened group.

**Table 4:** Baseline and 24-h respiratory parameters

Variables	MP variation rate ≤ 0% (MP-worsened, n = 173)	MP variation rate > 0% (MP-improved, n = 429)	*p*
Baseline tidal volume, mL/kg PBW	6.7 (5.9, 7.4)	7.1 (6.4, 7.9)	< 0.001
24-h tidal volume, mL/kg PBW	6.9 (6.2, 7.9)*	6.9 (5.9, 7.8)*	0.208
Baseline minute ventilation, L/min	8.4 (7.6, 9.4)	8.6 (7.7, 9.9)	0.115
24-h minute ventilation, L/min	9.0 (8.1, 10.2)*	7.5 (6.5, 8.5)*	< 0.001
Baseline PEEP, cmH_2_O	8 (6, 9)	7 (6, 8)	0.353
24-h PEEP, cmH_2_O	8 (7, 10)*	6 (5, 8)*	< 0.001
Baseline Cdyn, mL/cmH_2_O	31.7 (24.7, 37.5)	31.0 (25.3, 39.3)	0.63
24-h Cdyn, mL/cmH_2_O	30.9 (22.4, 39.3)	35.3 (27.6, 44.9)*	< 0.001
Baseline arterial pH	7.36 (7.29, 7.42)	7.39 (7.32, 7.45)	0.004
24-h arterial pH	7.43 (7.38, 7.47)*	7.44 (7.41, 7.48)*	0.024
Baseline PaCO2, mmHg	42.4 (37.6, 51.6)	38.9 (33.4, 44.3)	< 0.001
24-h PaCO2, mmHg	41.0 (37.7, 46.2)*	40.7 (37.0, 44.3)*	0.073
Baseline PaO_2_/FiO_2_ ratio, mmHg	123.6 (87.9, 167.0)	134.7 (98.0, 193.3)	0.006
24-h PaO_2_/FiO_2_ ratio, mmHg	212.8 (159.1, 280)*	245.0 (184.9, 294.3)*	0.003
Baseline FiO_2_, %	49 (39, 59)	45 (38, 55)	0.022
24-h FiO_2_, %	42 (36, 52)*	38 (32, 44)*	< 0.001
Baseline PaO_2_, mmHg	60.1 (49.0, 76.6)	64.2 (50.6, 80.0)	0.061
24-h PaO_2_, mmHg	86.9 (72.7, 104.0)*	86.5 (74.7, 104.1)*	0.811
Baseline ventilatory ratio	1.50 (1.30, 1.78)	1.42 (1.21, 1.68)	0.018
24-h ventilatory ratio	1.51 (1.33, 1.78)	1.25 (1.12, 1.41)*	< 0.001

IQR, interquartile range; PBW, predicted body weight; PEEP, positive end-expiratory pressure; Cdyn, dynamic compliance of respiratory system; PaO_2_, partial pressure of arterial oxygen; FiO_2_, fraction of inspired oxygen

^*^p < 0.05 comparing same parameters at baseline and 24-h by Wilcoxon signed-rank test

In both groups, FiO_2_ was downregulated, and PaO_2_ improved. PaCO_2_ was decreased in the MP-worsened group (42.4 mmHg [IQR 37.6–51.6 mmHg] vs 41.0 mmHg [IQR 37.7–46.2 mmHg]; *p* = 0.002) but increased in the MP-improved group (38.9 mmHg [IQR 33.4–44.3 mmHg] vs 40.7 mmHg [IQR 37.0–44.3 mmHg]; *p* < 0.001).

## Discussion

Our results showed that the 24-h MP variation rate, adjusted for confounders, was an independent risk factor for ICU mortality among ARF patients with elevated baseline MP. In addition, the main reason for a worsened MP value 24 h after admission was the increase in minute ventilation and PEEP.

Several clinical studies have shown that high MP was related to poor outcomes among ICU patients [6–9]. We consider the dynamic change of MP to be more informative than the MP at a certain time point because the former reflects a patient’s response to respiratory treatments, while the latter reflects only the static status of a patient. Response of MP to treatments could be more indicative of disease severity. This may explain why the 24-h MP variation rate remained an independent risk factor after adjusting for confounders, while the baseline MP and 24-h MP were not included in the present study (Table 2). Recently, Urner et al. [12] reported that cumulative exposure to higher intensities of mechanical ventilation was related to poor outcome, which was in line with our study. Decrease in MP within the first 24 h might be beneficial for patients with elevated MP on admission.

To further investigate the impact of lung conditions on our results, we used baseline MP as a stratification factor. Being the total energy load on lungs, the baseline MP, instead of APACHE II score or PaO_2_/FiO_2_ ratio, may better reflect the severity of lung injury on admission. Higher baseline MP was in accordance with higher APACHE II score and the ICU mortality (Table 3). We noticed that the prognostic values for baseline MP, 24-h MP and the MP variation rate were different among several subgroups of our database with various lung conditions on admission. The highest AUC-ROC for baseline MP appeared in unselected ICU patients, which may be due to a majority of patients with low baseline MP and low mortality, then it decreased with the increase in respiratory severity. The tendency of AUC-ROC for 24-h MP and the MP variation rate were the opposite: they tended to have a better performance in more severe cases (Table 3). It was not surprising that the poor prognostic value of the MP variation rate was seen among MP-unselected ARF patients. For those who did not suffer an injurious power at the start of ventilation, MP fluctuation within a relatively ‘safe’ range would contribute little to VILI, while for those who were exposed to higher MP on admission, a quick move to reduce MP would benefit the prognosis.

The dynamic changes in MP components among groups were simultaneously investigated. In the MP-improved group, minute ventilation was reduced, accompanied by a median PaCO_2_ increase above 40 mmHg (our quality control target). Both PEEP and FiO_2_ were downregulated, accompanied by improvements in respiratory compliance and oxygenation. In the MP-worsened group, the effort to reduce minute ventilation seemed to fail despite the use of more NMBAs. Since the 24-h PaCO_2_ was similar among groups, a higher minute ventilation could indicate a higher proportion of dead space estimated by the ventilatory ratio [20] and indicate a higher disease severity [22]. Notably, while the oxygenation was improved and FiO_2_ was downregulated on the first day, PEEP was somehow increased in the MP-worsened group, contributing to the rise of MP. This may raise an interesting question that whether patients in this group could benefit from lower PEEP and thus lower MP. The answer needs elaborate prospective study design.

A relatively low specificity and positive predictive value are explicable. Certain patients with ARF or acute respiratory distress syndrome (ARDS) experience deterioration of lung conditions in the first few days until the primary causes are controlled, during which if not exposed to hazardous MP long enough, they can still recover from acute lung injuries. Therefore, the positive predictive value could be improved in a population with severely high MP. Additionally, a cut-off value of approximately 20% for the MP variation rate would allow a fairly high negative predictive value of 80%, meaning that a quick action to reduce MP would indicate a higher probability of short-term survival. For example, this objective can be achieved by reducing minute ventilation from 10 to 8 L/min as long as the value of PaCO_2_ is within an acceptable range [23]. The control of tidal volume will create a more beneficial effect on MP since the airway pressure will decrease simultaneously, resulting in a larger drop in MP. This again emphasizes the importance of low tidal volumes, as has been strongly recommended in guidelines [14, 15], while the compliance rate should be enhanced [24].

We also noticed a difference in hemodynamic parameters: the MP-improved group had a lower central venous pressure (9.1 mmHg [IQR 7.6–10.4 mmHg] vs. 9.7 mmHg [IQR 8.0–11.1 mmHg]) and a tendency for more negative fluid balance (− 605 ml [IQR − 1492 to 91 ml] vs. − 386 ml [IQR − 1384 to 298 ml]; *p* = 0.082) 24 h after admission (Additional file 1: eTable 3). Although there was only a weak correlation between fluid balance and changes in respiratory compliance (Additional file 2: eFigure 1), we reasoned that the removal of extra fluid could contribute to the improvement of oxygenation and respiratory compliance. Evidence from a randomized controlled trial has shown that a conservative strategy of fluid management resulted in improved lung function and a shorter duration of mechanical ventilation in patients with acute lung injury [25].

Our study has certain limitations. First, this study was limited by its post hoc nature inherent to the retrospective analysis. The improvement in MP may reflect a positive response to treatment, yet it does not exclude a prompt correction of incorrect ventilator settings, although we tried to minimize the second possibility by collecting 12-h average value of ventilator parameters. Second, the nature of single-centre study restricted the generalization of our findings to other institutions and patient populations, although the inclusion of consecutive patients reduced the inclusion bias and reflected our practical settings. Third, the baseline MP in our cohort was lower compared with a previous retrospective study showing a possible cut-off MP value at 17 J/min [6]. But the dynamic MP in this study would underestimate the true MP value by replacing plateau pressure with peak inspiratory pressure, creating a bias of minute ventilation × resistive pressure/20 [26]. Fourth, concern may exist regarding whether the simplified formula for MP calculation is suitable for patients under pressure‑controlled mode [27]. Pressure‑controlled mode only accounted for a small part in this study (around 10%). We also considered the bias between the dynamic MP and the true MP to be minimal according to their geometrical relationship shown in Additional file 2: eFigure 2. Meanwhile, the MP variation rate is less affected by the bias of absolute MP values. Last but not least, the ROC analysis shows that MP variation has a poor predictive value, which may reflect that VILI accounts for only a small part of cause of death in our cohort.

## Conclusions

The 24-h MP variation rate was an independent risk factor for ICU mortality among patients with ARF and elevated MP. Early decreases in MP may provide prognostic benefits in this population. Tidal volume and minute ventilation seem to be the first and foremost targets in reducing MP.

## Supplementary Information

**Additional file 1: eTables.**

**Additional file 2: eFigures.**

## Data Availability

The data will be available upon reasonable requests. Yi Chi (e-mail: chiyi1@126.com) should be contacted if someone wants to request the data.

## Abbreviations

MP: Mechanical power
ARF: Acute respiratory failure
AUC-ROC: Areas under curve of receiver operating characteristic
VILI: Ventilator-induced lung injury
ICU: Intensive care unit
PEEP: Positive end-expiratory pressure
PaO_2_: Partial pressure of arterial oxygen
F_i_O_2_: Fraction of inspired oxygen
PaCO_2_: Partial pressure of arterial carbon dioxide
Pmean: Mean airway pressure
SpO_2_: Saturation of peripheral oxygen
PBW: Predicted body weight
NMBA: Neuromuscular blocking agents
ARDS: Acute respiratory distress syndrome
