# Supplementary material for: Twenty-four-hour mechanical power variation rate is associated with mortality among critically ill patients with acute respiratory failure: a retrospective cohort study
Source: BMC Pulm Med. 2021 Oct 25;21:331. doi: 10.1186/s12890-021-01691-4 (PMC8543779; doi:10.1186/s12890-021-01691-4)
Supplement: Supplementary file 1 — Additional file 1: eTables. [file 12890_2021_1691_MOESM1_ESM.docx]

**eTable 1: Amount of missing data for baseline and 24-hour variables included in the analysis.**

| **Variables** | **Missing data (n)** | **Missing data (%)** |
| --- | --- | --- |
| Baseline pH | 4 | 0.66 |
| Baseline PaCO2 | 4 | 0.66 |
| Baseline lactate | 4 | 0.66 |
| Baseline PaO2/FiO2 ratio | 6 | 1.00 |
| Baseline central venous pressure | 55 | 9.14 |
| Baseline ScvO2 | 57 | 9.47 |
| Baseline Pcv-aCO2 | 70 | 11.63 |
| 24-hour pH | 7 | 1.16 |
| 24-hour PaCO2 | 7 | 1.16 |
| 24-hour lactate | 8 | 1.33 |
| 24-hour PaO2/FiO2 ratio | 13 | 2.16 |
| 24-hour central venous pressure | 54 | 8.97 |
| 24-hour ScvO2 | 56 | 9.30 |
| 24-hour Pcv-aCO2 | 65 | 10.80 |

**eTable 2. Univariate and Multivariate Logistic Regression Analysis for ICU Mortality**

| **Variables** | **Univariate Logistic Regression** | | **Multivariate Logistic Regression**  **(+ 24-hour MP variation rate)** | |
| --- | --- | --- | --- | --- |
|  | **Odds Ratio (95% CI)** | ***p*** | **Odds Ratio (95% CI)** | ***p*** |
| **Baseline MP > 10 J/min (n=602)** |  |  |  |  |
| Age | 1.016 (1.011–1.021) | <0.001 | - | 0.132 |
| Sex (female as reference) | 0.687 (0.457–1.007) | 0.054 | - | 0.065 |
| APACHE II score | 1.095 (1.084–1.106) | <0.001 | 1.076 (1.048–1.104) | <0.001 |
| Admission type (surgical as reference) | 3.181 (2.734–3.702) | <0.001 | 2.322 (1.559–3.459) | <0.001 |
| Baseline PaO2/FiO2 ratio | 0.996 (0.995–0.997) | <0.001 | - | 0.268 |
| Baseline ventilatory ratio | 1.290 (1.083–1.536) | 0.004 | - | 0.941 |
| Prone positioning wihtin 24 hours | 1.909 (1.636–2.228) | <0.001 | - | 0.220 |
| NMBA usage wihtin 24 hours | 3.787 (3.000–4.780) | <0.001 | 2.124 (1.138–3.961) | 0.018 |
| **Baseline MP > 15 J/min (n=107)** |  |  |  |  |
| Age | 1.046 (1.017–1.076) | 0.002 | 1.049 (1.016–1.083) | 0.003 |
| Sex (female as reference) | 1.111(0.433–2.853) | 0.827 | **-** | 0.730 |
| APACHE II score | 1.053 (1.004–1.104) | 0.033 | **-** | 0.286 |
| Admission type (surgical as reference) | 1.271 (0.580–2.784) | 0.550 | - | 0.508 |
| Baseline PaO2/FiO2 ratio | 0.999 (0.992–1.006) | 0.830 | - | 0.454 |
| Baseline ventilatory ratio | 1.601 (0.723–3.546) | 0.246 | - | 0.788 |
| Prone positioning wihtin 24 hours | 0.758 (0.350–1.640) | 0.481 | - | 0.052 |
| NMBA usage wihtin 24 hours | 3.103 (1.210–7.957) | 0.018 | - | 0.174 |

MP = mechanical power; CI = confidence interval; APACHE = Acute Physiology and Chronic Health Evaluation; NMBA = neuromuscular blocking agent.

**eTable 3. Baseline and 24-hour Hemodynamic Parameters**

| **Variables** | **MP variation rate <= 0%**  **(MP-worsened, n = 173)** | **MP variation rate > 0%**  **(MP-improved, n = 429)** | ***p*** |
| --- | --- | --- | --- |
| Baseline heart rate, /min | 101 (90, 113) | 102 (91, 113) | 0.324 |
| 24-hour heart rate, /min | 96 (85, 108) * | 97 (88, 106) * | 0.966 |
| Baseline mean arterial pressure, mmHg | 85 (80, 91) | 88 (82, 94) | < 0.001 |
| 24-hour mean arterial pressure, mmHg | 87 (82, 94) * | 89 (83, 95) * | 0.028 |
| Baseline lactate, mmol/L | 1.9 (1.2, 5.3) | 2.2 (1.3, 5.5) | 0.353 |
| 24-hour lactate, mmol/L | 1.7 (1.1, 2.6) * | 1.4 (1.1, 2.1) * | 0.015 |
| Baseline peripheral perfusion index | 1.3 (0.7, 2.1) | 1.3 (0.7, 2.1) | 0.828 |
| 24-hour peripheral perfusion index | 1.4 (0.7, 2.1) | 1.4 (0.8, 2.1) | 0.456 |
| Baseline Pcv-aCO2, mmHg | 4.4 (2.9, 6.0) | 4.8 (3.1, 6.8) | 0.098 |
| 24-hour Pcv-aCO2, mmHg | 4.0 (2.7, 6.0) | 4.2 (2.8, 6.0) * | 0.522 |
| Baseline ScvO2, % | 76.3 (70.9, 82.8) | 74.5 (67.2, 80.6) | 0.021 |
| 24-hour ScvO2, % | 72.9 (65.9, 79.1) * | 73.2 (67.6, 79.8) | 0.369 |
| Baseline central venous pressure, mmHg | 10 (8, 11) | 10 (8, 11) | 0.406 |
| 24-hour central venous pressure, mmHg | 10 (8, 11) | 9 (8, 10) * | 0.004 |
| 24-hour fluid balance, mL | -386 (-1384, 298) | -605 (-1492, 91) | 0.082 |

IQR = interquartile range; PaCO2 = partial pressure of arterial carbon dioxide; FiO2 = fraction of inspired oxygen; Pcv-aCO2 = central venous-arterial carbon dioxide difference; ScvO2 = central venous oxygen saturation.

* *P* < 0.05 comparing baseline and 24-hour values of the same parameters by Wilcoxon signed-rank test.
