# Supplementary material for: Twenty-four-hour mechanical power variation rate is associated with mortality among critically ill patients with acute respiratory failure: a retrospective cohort study
Source: BMC Pulm Med. 2021 Oct 25;21:331. doi: 10.1186/s12890-021-01691-4 (PMC8543779; doi:10.1186/s12890-021-01691-4)
Supplement: Supplementary file 2 — Additional file 2: eFigures. [file 12890_2021_1691_MOESM2_ESM.pptx]

## Slide 1
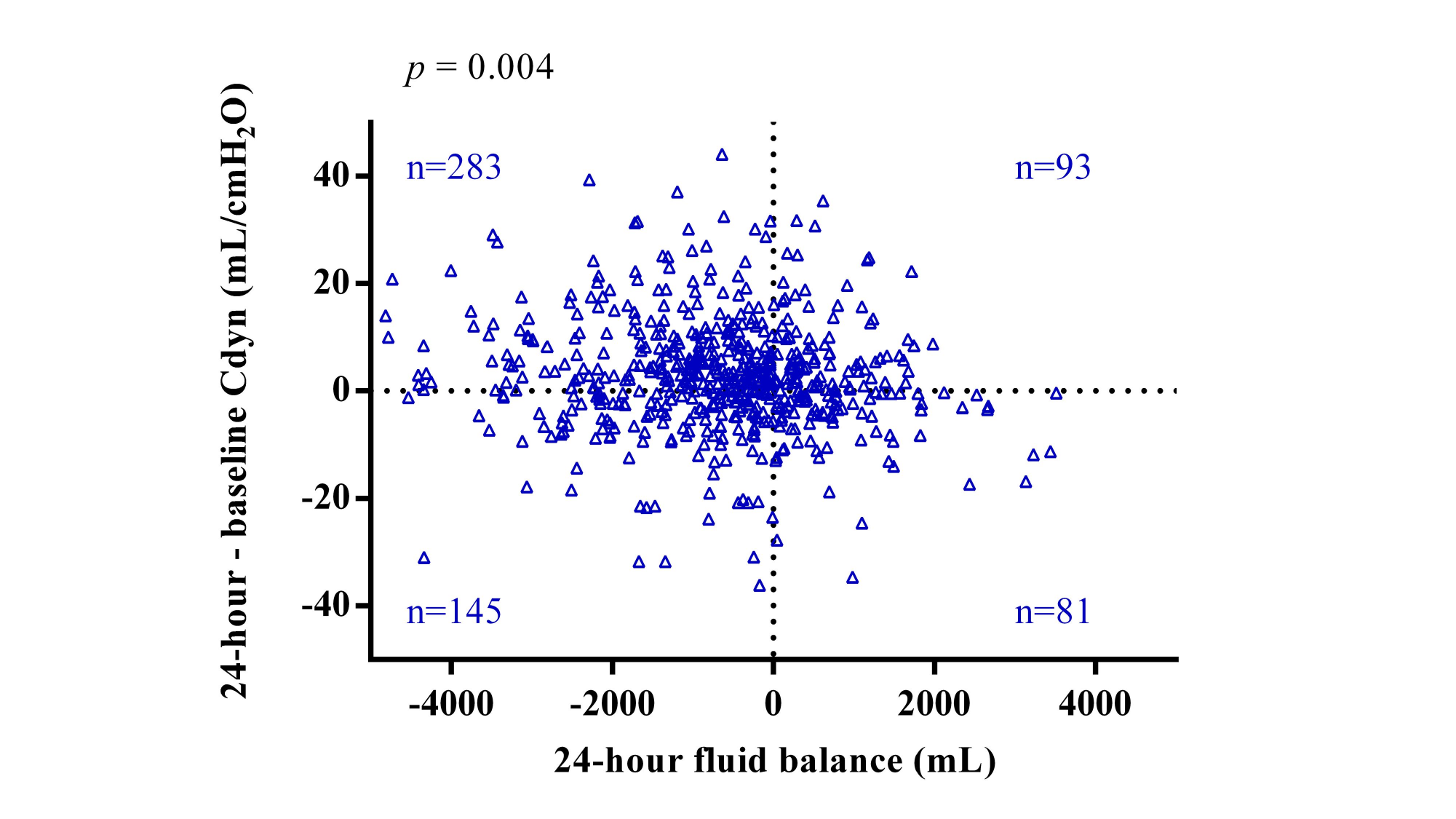

## Slide 2
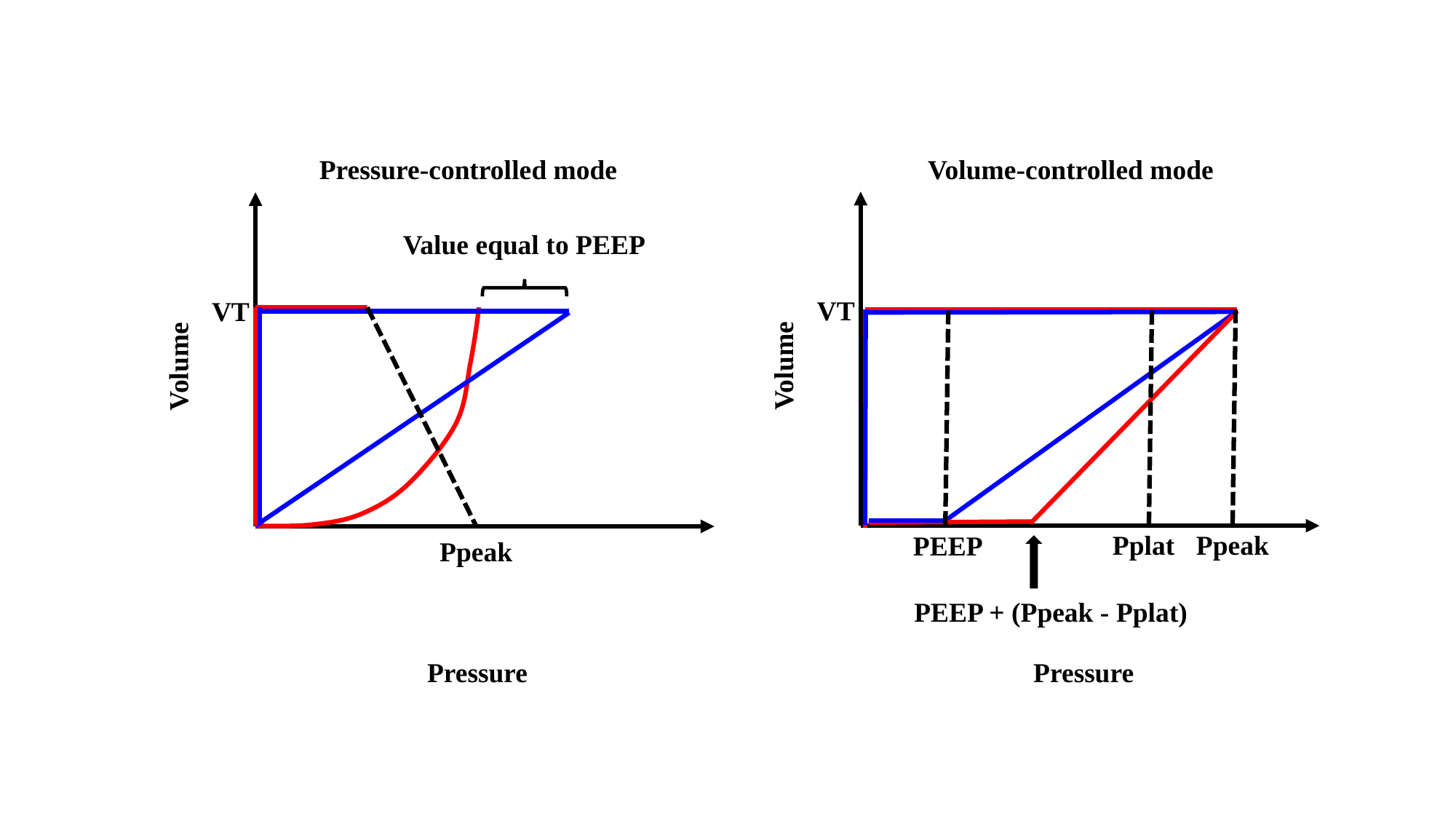

Pressure-controlled mode
Volume-controlled mode
Value equal to PEEP
VT
VT
Volume
Volume
Pplat
Ppeak
PEEP
Ppeak
PEEP + (Ppeak - Pplat)
Pressure
Pressure
